# The effects of prescribing varenicline on two‐year health outcomes: an observational cohort study using electronic medical records

**DOI:** 10.1111/add.14146

**Published:** 2018-02-20

**Authors:** Neil M. Davies, Gemma M. J. Taylor, Amy E. Taylor, Timothy Jones, Richard M. Martin, Marcus R. Munafò, Frank Windmeijer, Kyla H. Thomas

**Affiliations:** ^1^ Medical Research Council Integrative Epidemiology Unit University of Bristol Bristol UK; ^2^ Bristol Medical School, Population Health Sciences University of Bristol Bristol UK; ^3^ UK Centre for Tobacco and Alcohol Studies, School of Experimental Psychology University of Bristol Bristol UK; ^4^ National Institute for Health Research Collaboration for Leadership in Applied Health Research and Care West (NIHR CLAHRC West) University Hospitals Bristol NHS Foundation Trust Bristol UK; ^5^ Department of Economics University of Bristol Bristol UK

**Keywords:** CPRD, instrumental variables, mortality, myocardial infarction, nicotine replacement therapy, varenicline

## Abstract

**Aims:**

To investigate whether smokers prescribed varenicline had lower risks of serious ill‐health during the 4 years following treatment compared with those prescribed nicotine replacement therapy (NRT).

**Design:**

Observational cohort study of electronic medical records.

**Setting:**

A total of 370 UK general practices sampled from the Clinical Practice Research Datalink.

**Participants:**

A total of 126 718 patients aged 18 and over who were issued smoking cessation prescriptions between 1 September 2006 and 31 March 2014.

**Measurements:**

Our primary outcome was all‐cause mortality within 2 years of first prescription as indicated by linked Office of National Statistics data. Our secondary outcomes were cause‐specific mortality, all‐cause, cause‐specific hospitalization, primary care diagnosis of myocardial infarction or chronic obstructive pulmonary disease (COPD), body mass index and attendance rate to primary care within 2 years of first prescription. Risk differences and 95% confidence intervals were estimated by multivariable adjusted regression and propensity score matched regression. We used instrumental variable analysis to overcome residual confounding.

**Findings:**

People prescribed varenicline were healthier at baseline than those prescribed NRT in almost all characteristics, highlighting the potential for residual confounding. Our instrumental variable analysis results found that people prescribed varenicline had a similar risk of mortality at 2 years [risk difference per 100 patients treated = 0.67, 95% confidence interval (CI) = ‐0.11 to 1.46)] to those prescribed NRT, and there were similar rates of all‐cause hospitalization, incident primary‐care diagnoses of myocardial infarction and COPD. People prescribed varenicline subsequently attended primary care less frequently.

**Conclusions:**

Smokers prescribed varenicline in primary care in the United Kingdom do not appear to be less likely to die, be hospitalized or experience a myocardial infarction or chronic obstructive pulmonary disease during the following 2 years compared with smokers prescribed nicotine replacement therapy, but they gain more weight and attend primary care less frequently.

## Introduction

Meta‐analyses of randomized controlled trials (RCTs) have shown that five additional smokers will quit for every 100 smokers allocated to varenicline rather than nicotine replacement therapy (NRT) 1. These meta‐analyses have also shown that patients allocated to varenicline are unlikely to be at increased risk of short‐term neuropsychiatric or cardiovascular adverse events 2, 3. Both randomized trials and observational studies have suggested that patients prescribed varenicline are less likely to smoke in the short term 4, 5. These reductions in smoking should translate into health benefits in the years following treatment. However, we have relatively little evidence about the effects of prescribing varenicline in primary care on health outcomes in the years following treatment.

Experimental evidence about the effects of prescribing varenicline on health more than a year after prescription is limited, because trials of smoking cessation medications rarely have follow‐up greater than a year. Furthermore, the number of participants in clinical trials is relatively low and trial participants are unlikely to be representative of patients in primary care. In addition, adherence to treatment regimens in RCTs may not be comparable to everyday clinical care, and there may be selective outcome reporting of trials. Hence, trials provide relatively little evidence about the longer‐term effects of smoking cessation therapy on outcomes that are perhaps most important to smokers and clinicians, such as mortality, hospitalization and attendance to primary care. A recent observational study investigated the association of smoking cessation medications and cardiovascular and neuropsychiatric adverse events. However, it had a limited follow‐up of 6 months, did not investigate important outcomes such as mortality and hospitalization and suffered potentially from residual confounding 6.

Residual confounding is a major issue, because patients prescribed varenicline tend to be healthier than those prescribed NRT 7. Traditional epidemiological methods attempt to account for these differences by adjusting for or matching on observed differences using multivariable adjusted regression or propensity scores. These methods depend upon the assumption that there are no unmeasured differences between those prescribed varenicline and NRT. This assumption is implausible. There are many differences between patients that are difficult or impossible to measure in databases of electronic medical records, such as socio‐economic position, genetics and strength of addiction. An alternative approach is instrumental variable analysis, which exploits different assumptions 8. An instrumental variable is a naturally occurring variation in the data set that can be used as a proxy for intervention the patient received. Instruments are defined by three assumptions. First, the instrument must be associated with the likelihood of receiving an intervention. Secondly, the association of the instrument and the outcome must not be confounded. Thirdly, the instrument must only affect the outcome through its effect on the likelihood of receiving the intervention. Physicians’ preferences for one medication over another are a potential instrument for the medications they prescribe to their patients 9. Physicians who prefer varenicline are more likely to prescribe it. Physicians’ preferences for varenicline may not relate to their patients’ baseline comorbidities. Finally, physicians’ preferences should not affect their patients’ outcomes directly, independent of the prescription they issue. If these assumptions hold, then instrumental variable analysis would provide unbiased estimates of the causal effect of varenicline.

We have used these methods to provide evidence that varenicline causes patients to be more likely to quit smoking 5. In the current study, we investigated if smokers prescribed varenicline had lower risks of serious ill‐health compared to those prescribed NRT during the 4 years following treatment. We used three approaches to control for potential confounding: multivariable adjusted analyses, propensity score matched analyses and instrumental variable regression using data from the UK Clinical Practice Research Datalink (CPRD). Previous studies have used data from UK electronic medical records to investigate the effects of varenicline on neuropsychiatric outcomes 7. We add to these results by investigating the effects of smoking cessation therapy on mortality, outcomes in primary and secondary care and the frequency of primary care consultations.

## Methods

We conducted a prospective cohort study of all people prescribed varenicline or NRT in a sample of primary care practices in the United Kingdom. All hypotheses and analyses were pre‐specified and a study protocol was published 10. The CPRD's terms and conditions for accessing the data do not allow us to disseminate individual level patient data. However, researchers interested in accessing the data should contact the CPRD directly (enquires@cprd.com). The statistical code used to produce these results can be accessed online (https://github.com/nmdavies/varenicline-safety/). The study is registered at clinicaltrials.gov (NCT: NCT02681848) and the Open Science Framework (https://osf.io/g9ch2/).

### Study design and participants

The CPRD contains records from more than 15 million people, who are representative of the population of the United Kingdom 11. More than 98% of the UK population are registered with a general practitioner (GP). GPs provide access to National Health Service (NHS) services, and patients are limited to a choice of GP practice within their geographical area. Data on mortality and hospital admission from the UK Office of National Statistics and the Hospital Episodes Statistics data sets have been linked to all the practices included in this analysis. People who attended linked practices are similar to those from unlinked practices 7. We restricted our analysis to people with ‘acceptable’ records at practices which were designated as ‘up‐to‐standard’ by the CPRD 11. Acceptable records were those with information on the date of birth and sex, first registration date and no breaks in their registration. Up‐to‐standard practices provided continuous reporting of data and reported when their patients registered at and left the practice. We sampled all those prescribed NRT or varenicline by a GP at a linked practice after 1 September 2006 (when varenicline was introduced to the United Kingdom) until the end of follow‐up in the linked data on 31 March 2014. We included people who were issued their first prescription of either varenicline or NRT within the study period. To ensure that patients were starting a new course of treatment, and there had been sufficient registration period to define covariates, we excluded prescriptions issued to people who were prescribed smoking cessation medications in the 18 months prior to the start of study follow‐up. To ensure sufficient medical history to define covariates, we excluded prescriptions issued to people who registered at a practice within the previous year. We excluded people prescribed both NRT and varenicline on the same day. Supporting information, Fig. SA provides a flow‐chart of study inclusions and exclusions.

### Exposures

Exposure was the first prescription of either varenicline or NRT. Therefore, allocation to treatment remained the same throughout the entire follow‐up period regardless of subsequent prescriptions. We did this for two reasons: first, to ensure that our analysis was comparable to an intention‐to‐treat analysis in a randomized controlled trial 12, 13; and secondly, because treatment‐switching will be related strongly to the characteristics of participants, so that analyses which model treatment‐switching are likely to suffer from residual confounding. We estimated the effect of initially prescribing varenicline compared to initially prescribing NRT. We did not investigate another smoking cessation agent, bupropion, because it is rarely prescribed in the United Kingdom, and large randomized trials and systematic reviews have demonstrated that it is less efficacious than varenicline 4, 14.

### Outcomes

Our primary outcome was all‐cause mortality 2 years after the first prescription. In the Supporting information, we report differences in all outcomes at 3, 6, 9, 12 and 48 months. We chose the primary outcome and timing a priori because previous observational studies have suggested that differences in mortality occur within 2 years of smoking cessation 15. We also investigated the following secondary outcomes: mortality due to chronic lung disease (ICD‐10 codes = J40–44), lung cancer (ICD‐10 codes = C34), coronary heart disease (ICD‐10 codes = I21–25), pneumonia (ICD‐10 = J12–18), cerebrovascular disease (ICD‐10 = I60–69), diabetes (ICD‐10 = E10–14), external causes (ICD‐10 = V01‐Y98); all‐cause in‐patient hospitalization; in‐patient hospitalization for the same causes as mortality above (using the same ICD‐10 codes as for mortality); incident diagnosis of myocardial infarction, chronic obstructive pulmonary disease or diabetes (Read code lists available in the Supporting information); frequency of primary care consultations; and weight. The Read code lists were based on previously validated code lists 16.

### Potential confounders

We included the following potential confounders at baseline: sex; age; body mass index (BMI kg/m^2^); alcohol drug misuse, defined using Read codes; socio‐economic position measured by the index of multiple deprivation (IMD); an indicator for having more than five primary care consultations in the year before first prescription; indicators for year of prescription; any previous prescriptions of hypnotics; antipsychotics; antidepressants; diagnoses of: self‐harm; myocardial infarction; chronic obstructive pulmonary disease; any psychiatric disease; or serious comorbidities (Charlson Index) or any psychiatric disease. Product and Read codes lists for all potential confounders are available in the Supporting information. A total of 13.6% of patients were missing data on BMI and 0.1% were missing data on socio‐economic position. We imputed these values using the Imputation by Chained Equations (ICE) package in Stata; see Supporting information, Table S1 for comparison of imputed and complete samples 17.

### Data analysis

We used Stata version 14MP for all analyses. The statistical code used to produce these results can be accessed online (https://github.com/nmdavies/varenicline-safety).

### Multivariable adjusted analysis

We used logistic regression to estimate the associations of smoking cessation medications and the outcomes and report odds ratios (OR) and 95% confidence intervals (CI). We did not use Cox survival models as the proportional hazards assumption did not hold (based on the phtest command in Stata). Basic adjusted results account for sex, year of first prescription and age. The fully adjusted results also account for all potential confounders described above.

### Propensity score analysis

We created a propensity score using the command psmatch2 and the covariates described above 18. For this analysis, we replaced missing values of BMI and IMD at the mean and included indicator variables for missing values. We matched each patient prescribed varenicline with a patient with a similar propensity score using one‐to‐one nearest‐neighbour matching with no caliper. All patients prescribed varenicline had values of the propensity score within the range of propensity scores for the patients prescribed NRT. We estimated the ORs of outcomes within each follow‐up period and included the propensity score as a covariate. The propensity score results were consistent with and depend upon similar assumptions (conditional exchangeability) to the multivariable adjusted regression. Therefore, we present the propensity score analyses in the Supporting information.

### Instrumental variable analysis

To address residual confounding by unobserved comorbidities or other unmeasured confounders 7, we used instrumental variable analysis, which can provide unbiased estimates of the effects of treatment in the presence of unmeasured confounding 8, 19. Physicians’ prescribing preferences for specific medications have been proposed as potential instrumental variables for their prescriptions 9. Because the physicians’ preferences were not measured directly, we used the number of varenicline prescriptions they issued to their previous seven patients as a proxy. We pre‐specified seven prior prescriptions in our protocol on the basis of our previous study 20. Physicians who prescribed varenicline to their previous seven patients were classified as preferring varenicline and physicians who prescribed NRT to their previous seven patients were classified as preferring NRT.

For each of the outcomes, we created a set of binary outcomes indicating whether a patient had an event within 3, 6, 9, 12, 24 and 48 months of their first prescription. For each outcome, we censored patients who had an inadequate follow‐up. We used two‐stage least‐squares and additive structural mean models, which estimate mean and risk differences for continuous and binary outcomes, respectively 21. We tested the first instrumental variable assumption (instrument must be associated with the likelihood of receiving an intervention) using a partial *F*‐statistic to estimate the association of the physicians’ previous prescriptions (the instrument) and the prescription issued to their current patient (the exposure).

We tested the second instrumental variable assumption (whether the actual prescriptions and the instrumental variable were associated with observed covariates) using bias component plots (see Appendix for details) 22, 23. We restricted the instrumental variable analysis to physicians who issued more than 10 smoking cessation therapy prescriptions in our data set. This ensures that we had sufficient data to for each GPs. All standard errors and confidence intervals account for clustering of patients by GPs.

### Patient involvement

We presented our plans for the research and subsequently our results to the UK Centre for Alcohol and Tobacco Studies Smokers’ panel who provided feedback and recommendations on the research; for example, the need for more precise instrumental variable estimates of the effect on the primary outcome.

## Results

### Cohort characteristics

There were 126 718 patients who attended one of 370 practices who met the study inclusion criteria (Supporting information, Fig. S1). Of the included patients, 84 976 (67.1%) were prescribed NRT and 41 742 (32.9%) were prescribed varenicline (Table 1). The type of NRT issued to patients is shown in Supporting information, Table S2. Of the patients initially prescribed varenicline, 73% were issued more than one prescription for varenicline during the following 3 months. Twenty per cent of patients initially prescribed varenicline were issued more than 151 tablets during the following 3 months, the minimum number of tablets needed to adhere to a full course of treatment. Compared to people prescribed NRT, those prescribed varenicline were more likely to: be male, healthier, younger; and were less likely to: misuse alcohol or drugs, be from affluent areas, be less frequent primary care attendees, to have been prescribed hypnotics/anxiolytics, antipsychotics, antidepressants, statins, antihypertensives or diabetic medications and to have been diagnosed with self‐harm, myocardial infarction, chronic obstructive pulmonary disease, any psychiatric disease or other chronic disease (Charlson Index) (Table 1 and Supporting information, Fig. S2).

**Table 1:** Baseline characteristics of patients prescribed varenicline or nicotine replacement therapy. Data are number (%) of patients unless otherwise specified.

	Nicotine replacement therapy (n = 84 976)		Varenicline (n = 41 742)	
Male	39 285	46.2%	20 928	50.1%
Median age (SD)	46	24	44	19
Body mass index (SD)	25.5	7.1	25.7	6.6
Misuses alcohol	6199	7.3%	2086	5.0%
Misuses drugs	2484	2.9%	747	1.8%
Least deprived fifth of patients	11 209	13.2%	6427	15.4%
Most deprived fifth of patients	20 896	24.6%	9177	22.0%
Median number of primary care visits in prior year (SD)	6	8	5	6
Prescribed before 2009	38 568	45.4%	10 422	25.0%
Previous prescription of				
hypnotics/anxiolytic	17 419	20.5%	7114	17.0%
antipsychotic	16 918	19.9%	5975	14.3%
antidepressant	42 450	50.0%	17 895	42.9%
statins	16 534	19.5%	6217	14.9%
antihypertensive	17 608	20.7%	6974	16.7%
diabetic medication	7798	9.2%	2710	6.5%
Previous diagnosis of				
self‐harm	8846	10.4%	3614	8.7%
myocardial infarction	2624	3.1%	708	1.7%
chronic obstructive pulmonary disease	6837	8.0%	2516	6.0%
chronic disease (Charlson Index)	32 517	38.3%	13 277	31.8%
any psychiatric disease	41 275	48.6%	17 005	40.7%
Follow‐up time (years)	4.56	2.07	3.72	1.84

SD = standard deviation.

### Tests of plausibility of the instrumental variable assumptions

Physicians who prescribed varenicline to their previous patient were 24.5% (95% CI = 23.3 to 25.9%) more likely to prescribe varenicline to their subsequent patients. The instrument was the number of varenicline prescriptions issued by each patient's physician in their previous seven first‐time smoking cessation scripts. This explained a substantial proportion of the variation in prescribing: the partial *F*‐statistic, a measure of instrument strength, ranged from 4045 to 7775 24. These parameters indicate a strong instrument. The actual varenicline and NRT prescriptions were associated more strongly with the potential confounders than the instruments for these prescriptions. The bias component plots suggested that after accounting for the strength of the instrument, the bias components for the instrumental variable were smaller than those for the actual prescription for all covariates except BMI, number of prior GP visits and deprivation (Supporting information, Fig. S2 and Table S3).

### Difference in mortality and morbidity at 2‐year follow‐up

**Figure 1:**
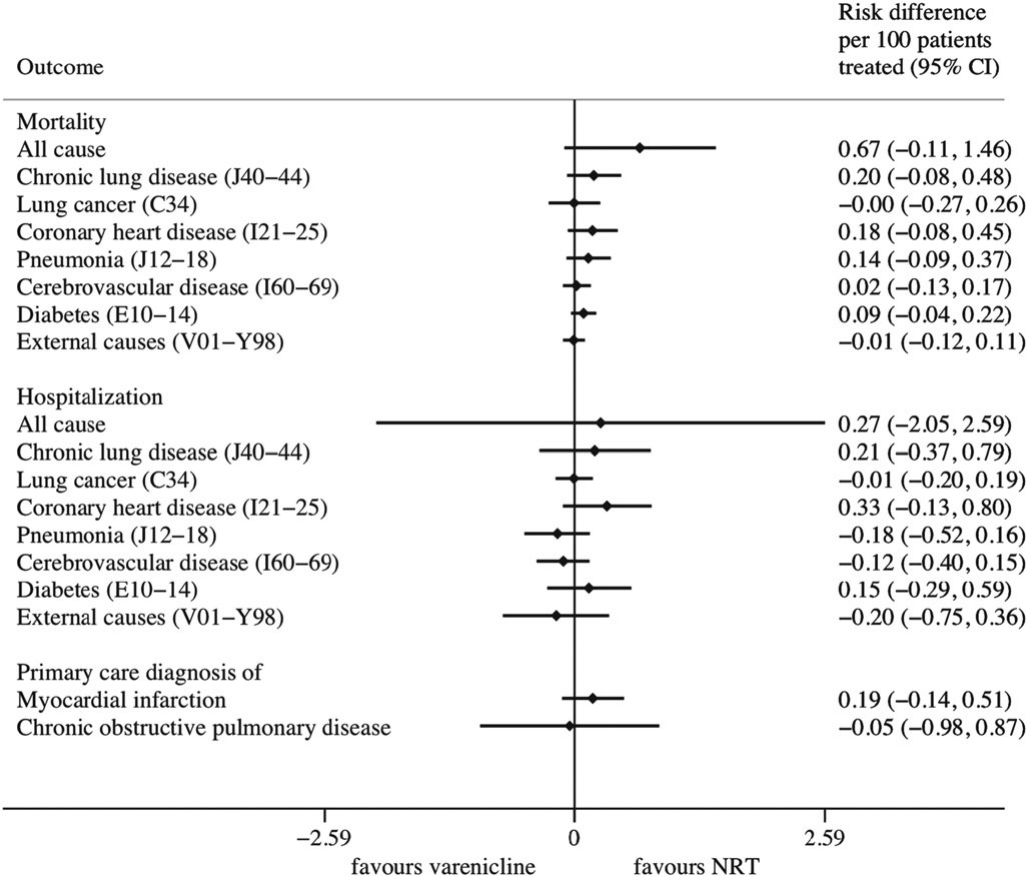
Instrumental variable estimates of the effects of prescribing varenicline versus nicotine replacement therapy on mortality and morbidity during the 2 years following first prescription. Confidence intervals allow for clustering between physicians. Instrumental variable results use seven prior prescriptions, and adjust for year of first prescription, gender and age

Prescribing varenicline rather than NRT did not improve outcomes during the 2 years following first prescription (Fig. 1). Specifically, varenicline increased the primary outcome, all‐cause mortality, relative to NRT, by 0.67 additional deaths (95% CI = –0.11 to 1.46%) per 100 people treated. There was little detectable difference in the effects of varenicline and NRT on cause‐specific mortality, all‐cause and cause‐specific hospitalization and rates of incident primary care diagnoses of myocardial infarction and chronic obstructive pulmonary disease.

### The effects of varenicline versus NRT during 4‐year follow‐up

**Figure 2:**
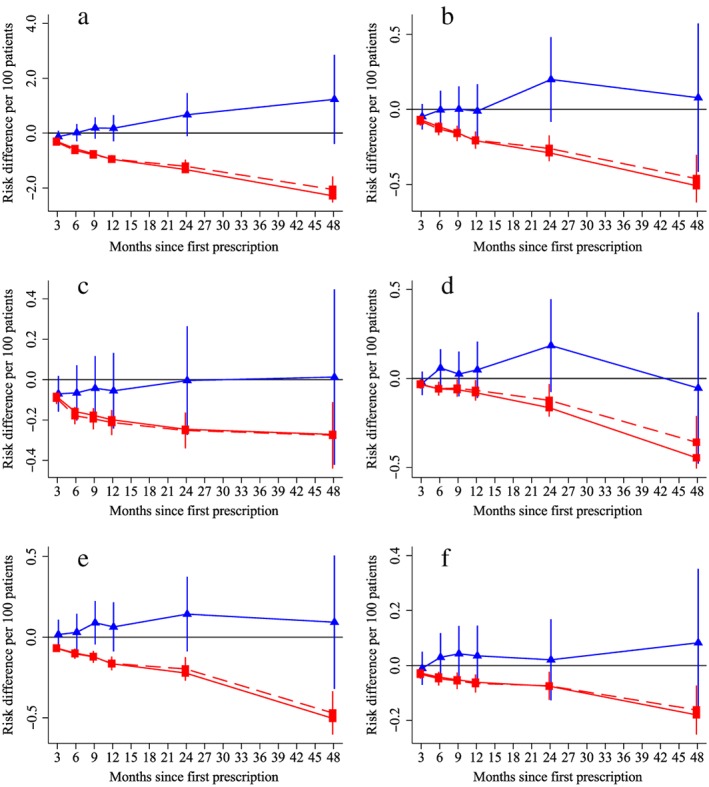
The effect of prescribing varenicline on (a) all‐cause, (b) chronic lung disease‐related (ICD J40–44), (c) lung cancer (ICD C34), (d) coronary heart disease (ICD I21–25), (e) pneumonia‐related (ICD J12–18) and (f) cerebrovascular disease (ICD I60–69)‐related mortality during the 4 years following first prescription as indicated via Office of National Statistics mortality records. Linear regression is indicated by 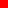, solid line is adjusted for basic confounders, dashed line adjusts for all confounders listed in Table 1 and the instrumental variable results are indicated by 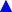; 95% confidence intervals indicated. Confidence intervals allow for clustering between physicians. The multivariable adjusted results adjust for all the covariates listed in Table 1. Instrumental variable results use seven prior prescriptions. [Colour figure can be viewed at http://wileyonlinelibrary.com]

The instrumental variable results found that varenicline, in comparison with NRT, was not associated with all‐cause or cause‐specific or mortality at any point throughout the 4‐year follow‐up (solid blue lines in Fig. 2a). In contrast, the multivariable adjusted regression results found that patients prescribed varenicline had lower all‐cause and cause‐specific mortality than those prescribed NRT (solid and dashed red lines in Fig. 2 for basic and fully adjusted analyses, respectively). These differences increased over time up to 4 years after the initial prescription.

**Figure 3:**
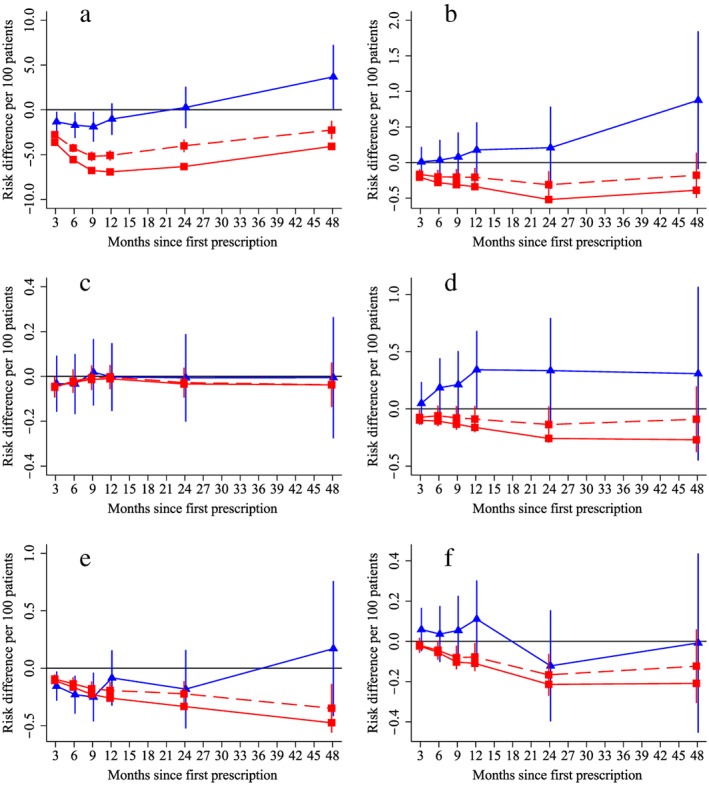
The effect of prescribing varenicline on (a) all‐cause, (b) chronic lung disease‐related (ICD J40–44), (c) lung cancer (ICD C34), (d) coronary heart disease (ICD I21–25), (e) pneumonia‐related (ICD J12–18) and (f) cerebrovascular disease (ICD I60–69)‐related hospital in‐patient admission during the 4 years following first prescription as indicated via Office of National Statistics death records. Linear regression is indicated by 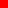, solid line is adjusted for basic confounders, dashed line adjusts for all confounders listed in Table 1 and the instrumental variable results are indicated by 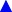; 95% confidence intervals indicated. Confidence intervals allow for clustering between physicians. The fully multivariable adjusted results adjust for all the covariates listed in Table 1. Instrumental variable results use seven prior prescriptions. [Colour figure can be viewed at http://wileyonlinelibrary.com]

The instrumental variable results found that prescribing varenicline rather than NRT reduced all‐cause in‐patient hospital admissions during the 9 months following the initial prescription (solid blue lines, Fig. 3a). However, by 4 years of follow‐up this effect had reversed. There was little evidence from the instrumental variable analysis that varenicline reduced rates of cause‐specific hospital admission compared to NRT (solid blue lines, Fig. 3b–f). In contrast, the multivariable results suggested that patients prescribed varenicline were substantially less likely to be hospitalized throughout the entire follow‐up (red lines in Fig. 3a). These differences attenuated after adjustment for observed confounders. The multivariable adjusted analysis suggested that patients prescribed varenicline rather than NRT generally had lower cause‐specific hospital admission for all outcomes except lung cancer (red lines Fig. 3b–f).

**Figure 4:**
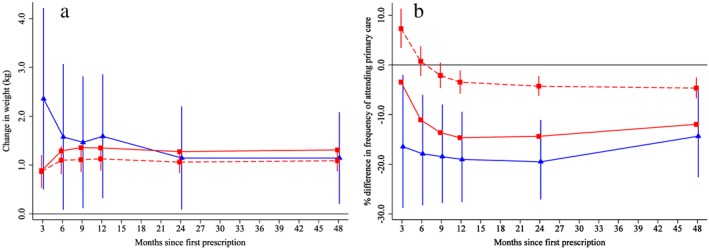
Effect of prescribing varenicline on (a) weight (kg) and (b) frequency of attendance to primary care during the 4 years following initial prescription. Linear regression is indicated by 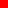, solid line is adjusted for basic confounders, dashed line adjusts for all confounders listed in Table 1 and the instrumental variable results are indicated by 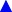; 95% confidence intervals indicated. [Colour figure can be viewed at http://wileyonlinelibrary.com]

**Figure 5:**
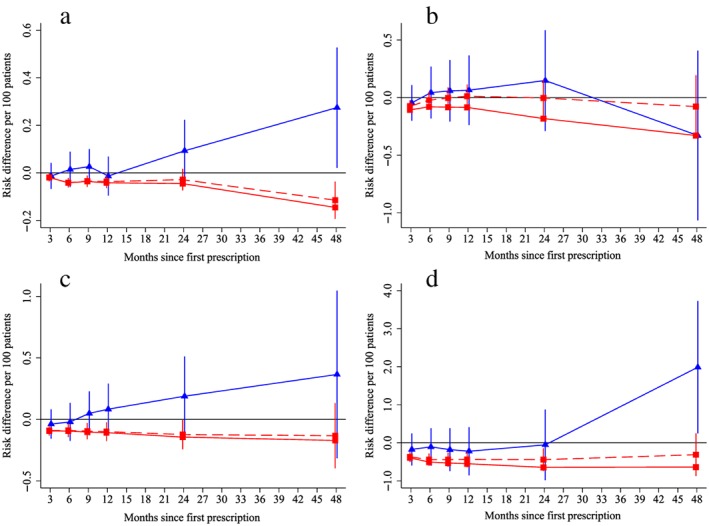
Effect of prescribing varenicline on diabetes‐related (ICD E10–14) (a) mortality and (b) in‐patient admission, primary‐care diagnosis of (c) myocardial infarction and (d) chronic obstructive pulmonary disease during the 4 years following initial prescription. Linear regression is indicated by 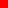, solid line is adjusted for basic confounders, dashed line adjusts for all confounders listed in Table 1 and the instrumental variable results are indicated by 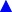; 95% confidence intervals indicated. Confidence intervals allow for clustering between physicians. The fully multivariable adjusted results adjust for all the covariates listed in Table 1. Instrumental variable results use seven prior prescriptions. [Colour figure can be viewed at http://wileyonlinelibrary.com]

Patients prescribed varenicline versus NRT gained 1.14 kg (95% CI = 0.08 to 2.20%) (Fig. 4a). This result was consistent across all analytical methods. Patients prescribed varenicline were also 19.5% (95% CI = 11.1 to 27.1%) less likely to attend primary care than those prescribed NRT (Fig. 4b**)**. After 4 years the instrumental variable results found that patients prescribed varenicline had a higher risk of diabetes‐related mortality, but not hospitalization (blue lines, Fig. 5a,b). This effect on diabetes may be related to weight gain 25. There was little evidence that varenicline lowered the risk of incident primary care diagnoses of myocardial infarction or chronic obstructive pulmonary disease (blue lines, Fig. 5c,d).

Propensity score matched regression results are available in the appendix (Supporting information, Tables S4, S5). The propensity score results were similar to the multivariable adjusted results. The distributions of the propensity scores and the balance of covariates before and after matching can be seen in Supporting information, Figs S3, S4 and Table S6.

## Discussion

We found little evidence that patients prescribed varenicline versus NRT had a lower risk of mortality, hospitalization or primary care diagnosis of myocardial infarction or chronic obstructive pulmonary disease 2 years after first prescription. Both multivariable adjusted and instrumental variable regression suggested that patients prescribed varenicline attended primary care less frequently during the 2 years following the first prescription.

Patients prescribed varenicline were healthier than those prescribed NRT at baseline in almost all ways we could measure. It is, therefore, likely that there are differences in terms of unobserved characteristics (i.e. genetics, income, education, strength of addiction and motivation to quit). Hence, the results from multivariable adjusted and propensity score matched regression are unlikely to reflect the causal effect of prescribing varenicline, as both approaches depend upon the assumption that there are no unmeasured confounders. This may explain why we found implausibly large short‐term differences in all‐cause and cause‐specific mortality in the multivariable adjusted and propensity score regression. In contrast, the instrumental variable analysis suggested little short‐term effect of prescribing varenicline versus NRT on mortality; these results are more robust to confounding by unobserved patient characteristics (healthy user bias). We found consistent evidence from all approaches that patients prescribed varenicline subsequently experienced increases in weight and attended primary care less frequently. This may suggest that varenicline affects weight and GP attendance, potentially via its effects on smoking cessation. In contrast, the other outcomes we investigated may not be affected as strongly by varenicline (and smoking cessation) as implied by the partially or fully or multivariable adjusted differences. It is conceivable that a similar bias afflicts results from other observational studies of the effect of smoking cessation 1.

### Strengths and limitations

A key strength of our study is that we used three different statistical techniques to address residual confounding, multivariable adjusted, propensity score and instrumental variable regression. Our multivariable adjusted results could suffer from residual confounding and healthy user bias. During this period there were safety warnings and scientific papers which suggested that patients prescribed varenicline might be at increased risk of neuropsychiatric and cardiovascular adverse events. This could explain why patients prescribed varenicline were healthier at baseline. To overcome this, we used three different approaches to control for differences between patients that depend upon distinct assumptions. While the multivariable adjusted association with mortality was large and attenuated only modestly after adjustment for observed confounders, the instrumental variable results suggested little short‐term reduction in mortality. Therefore, the association of varenicline prescriptions and mortality observed in the multivariable and propensity score regression analyses is likely to be due to residual confounding. The instrumental variable results could suffer from bias if there are unobserved confounders affecting the instrument, the physicians’ preferences and the outcome 22. We investigated this in terms of observed characteristics of the patients and found that the instruments tended to much less associated with the observed confounders than the actual prescription the patient received. This is consistent with the instrumental variable results being less biased.

Patients prescribed smoking cessation medication may receive other cessation support (e.g. one‐on‐one or group smoking cessation sessions). If patients prescribed varenicline have a different likelihood of receiving these interventions compared to those prescribed NRT, then it could bias our observational results. The CPRD does not contain detailed information on referrals to smoking cessation services. Treatment differences by prescription issued would not necessarily bias our instrumental variable estimates. However, if physicians who preferred varenicline were also more likely to prescribe other interventions, this could bias our instrumental variable results positively.

Only a proportion (73%) of patients prescribed varenicline received more than one prescription in the 3‐month treatment period, and only 20% received sufficient tablets to complete a course of treatment. This means that our results may underestimate the effects of complying with treatment (as opposed to the effects of being prescribed treatment). While the effect of prescribing treatment is of more interest to clinicians, the effect of complying is perhaps of most interest to patients. However, it is difficult to estimate the effects of compliance, independently of treatment initiation, because compliance is non‐random.

A strength of our study is that we published a protocol and pre‐specified our primary outcome 10. A potential limitation of our study is that we had no data on patients’ use of over‐the‐counter NRT. This means that patients observed in our study may have attempted to quit previously without attending primary care. However, as varenicline is only available via prescription in the United Kingdom, we are likely to have observed all uses of varenicline. However, smokers who purchase over‐the‐counter NRT are unlikely to be representative of patients who attend primary care for smoking cessation advice. Therefore, even if we had the data, users of over‐the‐counter NRT are unlikely to be an appropriate control group for patients prescribed varenicline. Furthermore, our results relate to the initial decision to prescribe either varenicline or NRT, which may not reflect the effects of longer‐term prescribing. Our results are therefore likely to be representative of the treatment decisions and outcomes of patients who consult with physicians about smoking cessation.

Our outcomes were derived from validated code lists and algorithms. However, it is possible that not all adverse events are coded in the patients’ electronic medical records. While this is a concern for the primary care diagnoses, there is unlikely to be missing information for hospital admissions or all‐cause mortality. These data come from linked national administrative records and validation studies have found that they record outcomes accurately [26].

A limitation of our study is that we only followed patients for up to 4 years. This means we were not able to test whether prescribing varenicline affected long‐term health outcomes. Many of the adverse health conditions associated with tobacco consumption are the result of many years of cumulative exposure to tobacco smoke (e.g. lung cancer). Future studies could follow‐up patients for longer using routine health records and potentially use recently proposed instrumental variable estimators for survival analysis 27.

### Comparison with other studies

Our instrumental variable results suggested that varenicline was unlikely to have large effects on risk of coronary heart disease mortality in the year following first prescription (risk difference (RD) per 100 patients treated = 0.05%, 95% CI = –0.11 to 0.21%; hospitalization due to coronary heart disease (RD = 0.34%, 95% CI = 0.00 to 0.68%); or primary care diagnoses of myocardial infarction (RD = 0.08%, 95% CI = –0.13 to 0.29%). This means we can exclude a greater than 1 in 147 increase in risk of coronary heart disease mortality; this is likely to be smaller than the longer‐term reduction in risk of cardiovascular events caused by quitting smoking. In comparison, a network meta‐analysis of randomized trials found that participants allocated to varenicline had similar rates of major adverse cardiovascular events (RD = −0.06%, 95% CI = –0.14 to 0.17%, assuming 0.2 events per 100 allocated to NRT) 28. The CATS trial (Cardiac Assessments Following Different Treatments of Smoking Cessation Medications in Subjects With and Without Psychiatric Disorders), an extension to the EAGLES study (Evaluating Adverse Events in a Global Smoking Cessation Study)**,** found that participants allocated to varenicline had similar risk of major adverse cardiovascular events to those allocated to NRT in the 6–12 months following treatment (RD = −0.15%, 95% CI = –0.44 to 0.14%) 29. However, the follow‐up to this study was relatively short. The multivariable adjusted estimates of the effect of varenicline on rates of primary care diagnosis of myocardial infarction were similar in magnitude to a previous observational study using electronic medical records 6.

### Generalizability

The CPRD is representative of the United Kingdom, therefore our results are likely to reflect the association of smoking cessation medications and adverse outcomes in primary care in the United Kingdom. Consequently, the individuals in our study are likely to have more comorbidity and be more representative of the general population than participants of randomized controlled trials. Whereas only seven (0.09%)of 8058 participants in the EAGLES trial died within 24 weeks of randomization, 740 (0.72%) of 103 162 patients in the CPRD died within 6 months of first prescription. Our instrumental variable results estimate a so‐called ‘local average treatment effect’, under the assumption of a monotonic effect of the instrument on the likelihood of being prescribed varenicline 30. This assumption requires that a patient who is prescribed varenicline by a physician who prefers NRT would also receive varenicline had they attended a physician who preferred varenicline, and vice versa. This is the average effect of varenicline in patients whose treatment was affected by their physician's preferences. These estimates may not be valid for other patients; for example, patients who have strong contraindications for varenicline.

Patients who were prescribed varenicline as part of their everyday clinical care experienced similar rates of adverse health outcomes to patients prescribed NRT during the 4 years following treatment. There were few differences in rates of all‐cause and cause‐specific mortality or hospitalization. However, consistent with previous studies 31, patients prescribed varenicline gained more weight and had a higher risk of diabetes. These results raise questions about the effects of prescribing varenicline on health in the years following treatment. Patients who quit smoking may need additional health monitoring in the years following cessation.

### Study registration

NCT02681848.

### Data access

The statistical code used to produce these results can be accessed here: (https://github.com/nmdavies/varenicline-safety). The data set used in this study was extracted from the CPRD, which can be accessed by contacting the CPRD kc@cprd.com.

### Ethics

This study was approved by the CPRD's Independent Scientific Advisory Committee (ISAC), protocol number 15_107.

## Declaration of interests

All authors have completed the ICMJE uniform disclosure form at http://www.icmje.org/coi_disclosure.pdf. We declare the following interests: M.R.M. reports grants from Pfizer, grants from Rusan, and non‐financial support from GlaxoSmithKline, outside the submitted work; A.E.T. reports a grant from the Global Research Awards for Nicotine Dependence, which is an Independent Competitive Grants Program supported by Pfizer. R.M.M. was a member of the Independent Scientific Advisory Committee of the Medicines and Healthcare products Regulatory Agency which approves applications for CPRD studies. All other authors report no other relationships or activities that could appear to have influenced the submitted work.

## Supporting information


**Figure S1** Flow‐chart of the number (n) of patients and records assessed for eligibility and reasons for exclusion.
**Figure S2** Relative linear regression and instrumental variable bias component terms instrumental variable results are indicated by 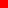 and 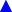, respectively; 95% confidence intervals plotted. The patients’ actual prescriptions were associated more strongly with the measured covariates than the proposed instrument, even after taking into account the instrument strength. This suggests that the instrumental variable estimates are likely to be less biased than the linear regression estimates.
**Figure S3** Distributions of propensity scores by treatment prescribed before matching.
**Figure S4** Distributions of propensity scores by treatment prescribed after matching.
**Table S1** Distributions of imputed characteristics in the imputation data sets and in observed data (i.e. without imputation) n eligible = 126 718.
**Table S2** Type of nicotine replacement therapy prescribed to patients.
**Table S3** Estimated linear regression and instrumental variable bias components.
**Table S4** Adjusted relative outcome rate among patients treated with varenicline or nicotine replacement therapy using propensity score methods. Follow‐up at 3, 6, 9, 12, 24 and 48 months.
**Table S5** Adjusted relative outcome frequency among patients treated with varenicline or nicotine replacement therapy using propensity score methods. Follow‐up at 3, 6, 9, 12, 24 and 48 months. Non‐imputed data.
**Table S6** Means and bias of baseline covariates before and after propensity score matching.
