# Supplementary material for: The effects of prescribing varenicline on two‐year health outcomes: an observational cohort study using electronic medical records
Source: Addiction. 2018 Feb 20;113(6):1105–16. doi: 10.1111/add.14146 (PMC5969097; doi:10.1111/add.14146)
Supplement: Supplementary file 1 — Figure S1 Flow‐chart of the number (n) of patients and records assessed for eligibility and reasons for exclusion. Figure S2 Relative linear regression and instrumental variable bias component terms instrumental variable results are indicated by and , respectively; 95% confidence intervals plotted. The patients’ actual prescriptions were associated more strongly with the measured covariates than the proposed instrument, even after taking into account the instrument strength. This suggests that the instrumental variable estimates are likely to be less biased than the linear regression estimates. Figure S3 Distributions of propensity scores by treatment prescribed before matching. Figure S4 Distributions of propensity scores by treatment prescribed after matching. Table S1 Distributions of imputed characteristics in the imputation data sets and in observed data (i.e. without imputation) n eligible = 126 718. Table S2 Type of nicotine replacement therapy prescribed to patients. Table S3 Estimated linear regression and instrumental variable bias components. Table S4 Adjusted relative outcome rate among patients treated with varenicline or nicotine replacement therapy using propensity score methods. Follow‐up at 3, 6, 9, 12, 24 and 48 months. Table S5 Adjusted relative outcome frequency among patients treated with varenicline or nicotine replacement therapy using propensity score methods. Follow‐up at 3, 6, 9, 12, 24 and 48 months. Non‐imputed data. Table S6 Means and bias of baseline covariates before and after propensity score matching. [file ADD-113-1105-s001.docx]

# The Effects of Prescribing Varenicline on Two-year Health Outcomes: an Observational Cohort Study Using Electronic Medical Records

**Supplementary materials**

Neil M Davies*^1,2^, Gemma MJ Taylor^1,3^, Amy E Taylor^1,3^, Tim Jones^4^_,_ Richard M Martin^1,2,4^, Marcus R Munafò^1,3^, Frank Windmeijer,^1,5^ and Kyla H Thomas.^2^

^1^ Medical Research Council Integrative Epidemiology Unit at the University of Bristol, BS8 2BN, United Kingdom.

^2^ Bristol Medical School, Population Health Sciences, University of Bristol, Barley House, Oakfield Grove, Bristol, BS8 2BN, United Kingdom.

^3^ UK Centre for Tobacco and Alcohol Studies, School of Experimental Psychology, University of Bristol, 12a Priory Road, Bristol, BS8 1TU.

^4^ National Institute for Health Research Collaboration for Leadership in Applied Health Research and Care West (NIHR CLAHRC West) at University Hospitals Bristol NHS Foundation Trust, 9^th^ Floor Whitefriars, Lewins Mead, Bristol, BS1 2NT

^5^ Department of Economics, University of Bristol, 2 Priory Road Complex, Bristol BS8 1TU, United Kingdom.

Web appendix: Supplementary material

Table of Contents

[The Effects of Prescribing Varenicline on Two-year Health Outcomes: an Observational Cohort Study Using Electronic Medical Records 1](#_Toc501011332)

[Supplementary Figure S1: Flow chart of the number (n) of patients and records assessed for eligibility and reasons for exclusion 4](#_Toc501011333)

[Supplementary Figure S2: Relative linear regression and instrumental variable bias component terms instrumental variable results are indicated by ■ and ▲ respectively. 95% confidence intervals plotted. The patients actual prescriptions were more strongly associated with the measured covariates than the proposed instrument, even after taking into account the instrument strength. This suggests that the instrumental variable estimates are likely to be less biased than the linear regression estimates. 5](#_Toc501011334)

[Supplementary Figure S3: Distributions of propensity scores by treatment prescribed before matching. 6](#_Toc501011335)

[Supplementary Figure S4: Distributions of propensity scores by treatment prescribed after matching. 7](#_Toc501011336)

[Supplementary Table S1: Distributions of imputed characteristics in the imputation datasets and in observed data (i.e. without imputation) N eligible=126,718 8](#_Toc501011337)

[Supplementary Table S2: Type of nicotine replacement therapy prescribed to patients 9](#_Toc501011338)

[Supplementary Table S3: Estimated linear regression and instrumental variable bias components 10](#_Toc501011339)

[Supplementary Table S4: Adjusted Relative Outcome Rate Among Patients Treated With Varenicline or Nicotine Replacement Therapy Using Propensity score Methods. Follow-up at 3, 6, 9, 12, 24, 48 Months. 11](#_Toc501011340)

[Supplementary Table S5: Adjusted Relative Outcome Frequency Among Patients Treated With Varenicline or Nicotine Replacement Therapy Using Propensity score Methods. Follow-up at 3, 6, 9, 12, 24, 48 Months. Non-imputed data. 13](#_Toc501011341)

[Supplementary Table S6: Means and bias of baseline covariates before and after propensity score matching. 14](#_Toc501011342)

## Supplementary Figure S1: Flow chart of the number (n) of patients and records assessed for eligibility and reasons for exclusion

## Supplementary Figure S2: Relative linear regression and instrumental variable bias component terms instrumental variable results are indicated by ■ and ▲ respectively. 95% confidence intervals plotted. The patients actual prescriptions were more strongly associated with the measured covariates than the proposed instrument, even after taking into account the instrument strength. This suggests that the instrumental variable estimates are likely to be less biased than the linear regression estimates.

Notes: Confidence intervals allow for clustering between physicians. All covariates aside from sex adjust for year, sex and age. Instrumental variable defined using seven prior prescriptions.

## Supplementary Figure S3: Distributions of propensity scores by treatment prescribed before matching.

**
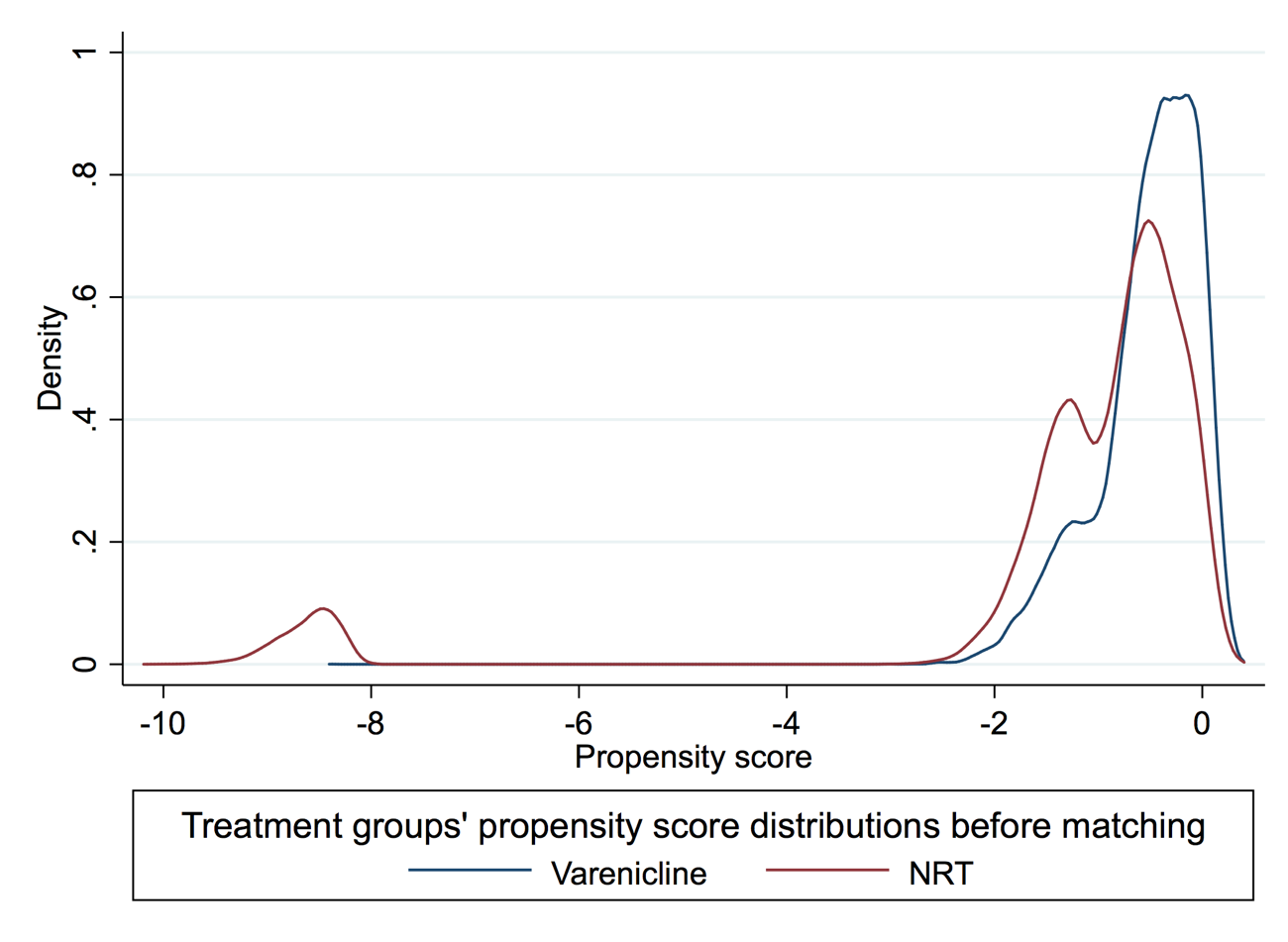
**

## Supplementary Figure S4: Distributions of propensity scores by treatment prescribed after matching.

**
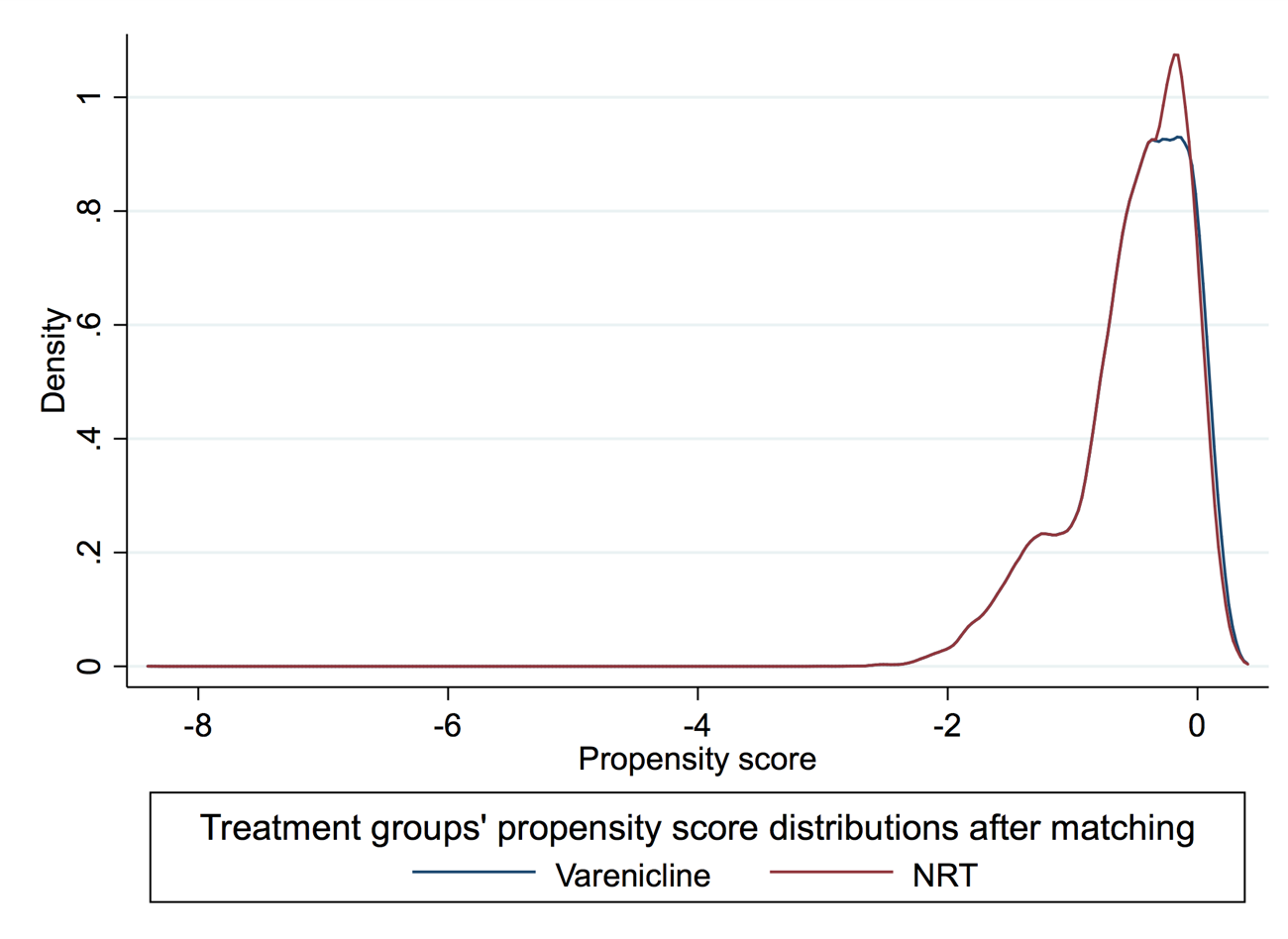
**

## Supplementary Table S1: Distributions of imputed characteristics in the imputation datasets and in observed data (i.e. without imputation) N eligible=126,718

| Imputed variable | % of data | Distribution Mean (SE) | |  |  |  |
| --- | --- | --- | --- | --- | --- | --- |
|  | imputed | Imputed | | Observed (with no missing) | | N |
|  |  | dataset | | dataset | |  |
|  |  | Mean | Standard error | Mean | Standard error |  |
| Body mass index | 13.6 | 26.4 | 0.02 | 26.5 | 0.02 | 109,542 |
| Index of multiple deprivation | 0.1 | 11.6 | 0.02 | 11.6 | 0.02 | 126,591 |

## Supplementary Table S2: Type of nicotine replacement therapy prescribed to patients

| Formulation type | Number prescribed | Proportion |
| --- | --- | --- |
| Transdermal patch | 65,143 | 42.60 |
| Tablets | 26,503 | 17.33 |
| Inhalation vapour | 25,100 | 16.41 |
| Film Coated Tablets | 17,607 | 11.51 |
| Medicated chewing-gum | 7,482 | 4.89 |
| Lozenge | 6,015 | 3.93 |
| Mouth Spray | 2,285 | 1.49 |
| Sublingual tablet | 1,777 | 1.16 |
| Spray | 966 | 0.63 |
| Not applicable | 29 | 0.02 |
| Orodispersible Film | 19 | 0.01 |
| Liquid | 1 | 0.00 |

Notes: Number of prescriptions indicated. Some patients were prescribed multiple types of nicotine replacement therapy for their first prescription.

## Supplementary Table S3: Estimated linear regression and instrumental variable bias components

|  |  | Difference per 100 patients treated (95% Confidence intervals) | | Test for |
| --- | --- | --- | --- | --- |
| Covariate | N | Ordinary least squares | Instrumental variables | heterogeneity |
| Male sex | 124,397 | 3.56 (2.91 to 4.21) | 0.93 (-1.72 to 3.57) | 0.041 |
| Median age (SD) | 124,397 | -224.59 (-246.21 to -202.96) | -152.99 (-234.67 to -71.31) | 0.065 |
| Body mass index (SD) | 107,582 | 1.43 (-6.05 to 8.90) | -6.99 (-37.60 to 23.61) | 0.573 |
| Alcohol misuse | 124,397 | -2.60 (-2.92 to -2.29) | -1.04 (-2.31 to 0.23) | 0.011 |
| Drug misuse | 124,397 | -1.46 (-1.65 to -1.27) | -1.01 (-1.80 to -0.21) | 0.248 |
| Least deprived fifth of patients | 124,273 | -2.81 (-4.18 to -1.44) | -1.39 (-7.05 to 4.27) | 0.531 |
| Most deprived fifth of patients | 124,273 | 2.13 (1.32 to 2.94) | 4.28 (0.94 to 7.61) | 0.118 |
| Median number of GP visits in year before treatment (SD) | 124,397 | -150.71 (-162.89 to -138.52) | -131.89 (-183.24 to -80.54) | 0.401 |
| Previous use of |  |  |  |  |
| Hypnotics/Anxiolytic | 124,397 | -3.41 (-3.95 to -2.86) | -1.50 (-3.73 to 0.74) | 0.070 |
| Antipsychotic | 124,397 | -4.88 (-5.41 to -4.35) | -2.68 (-4.79 to -0.58) | 0.026 |
| Anti-depressant | 124,397 | -6.26 (-6.98 to -5.54) | -1.83 (-4.61 to 0.96) | 0.001 |
| Statins | 124,397 | -2.75 (-3.21 to -2.29) | -0.81 (-2.71 to 1.09) | 0.032 |
| Anti-hypertensive | 124,397 | -2.31 (-2.77 to -1.84) | -1.44 (-3.36 to 0.48) | 0.350 |
| Diabetic medication | 124,397 | -2.46 (-2.80 to -2.13) | -0.30 (-1.76 to 1.16) | 0.002 |
| Previous diagnosis of |  |  |  |  |
| Self-harm | 124,397 | -2.15 (-2.53 to -1.76) | -1.17 (-2.79 to 0.45) | 0.214 |
| Myocardial infarction | 124,397 | -1.12 (-1.30 to -0.94) | 0.18 (-0.60 to 0.97) | 0.001 |
| Chronic obstructive pulmonary disease | 124,397 | -0.81 (-1.12 to -0.49) | -0.60 (-1.91 to 0.71) | 0.744 |
| Chronic disease (Charlson index) | 124,397 | -4.93 (-5.59 to -4.27) | -0.71 (-3.30 to 1.89) | 0.001 |

Notes: Bias components estimated via GMM. The null hypothesis of the heterogeneity test is that there is no difference between the linear regression and the linear regression bias terms.

## Supplementary Table S4: Adjusted Relative Outcome Rate Among Patients Treated With Varenicline or Nicotine Replacement Therapy Using Propensity score Methods. Follow-up at 3, 6, 9, 12, 24, 48 Months.

|  |  | Number of | Number of | Odds-ratio |
| --- | --- | --- | --- | --- |
|  |  | events | patients | (95% Confidence interval) |
| Mortality: |  |  |  |  |
| All cause | 3 | 192 | 80362 | 0.41 (0.30 to 0.57) |
|  | 6 | 362 | 77343 | 0.41 (0.32 to 0.52) |
|  | 9 | 508 | 74102 | 0.46 (0.38 to 0.56) |
|  | 12 | 684 | 70566 | 0.49 (0.42 to 0.57) |
|  | 24 | 1172 | 56184 | 0.68 (0.60 to 0.77) |
|  | 48 | 1389 | 27871 | 0.72 (0.64 to 0.81) |
| Cardiovascular disease | 3 | 52 | 80222 | 0.44 (0.24 to 0.81) |
|  | 6 | 111 | 77092 | 0.48 (0.32 to 0.72) |
|  | 9 | 152 | 73746 | 0.58 (0.42 to 0.81) |
|  | 12 | 193 | 70075 | 0.62 (0.46 to 0.83) |
|  | 24 | 351 | 55363 | 0.75 (0.61 to 0.94) |
|  | 48 | 406 | 26889 | 0.75 (0.61 to 0.91) |
| Respiratory disease | 3 | 74 | 80244 | 0.27 (0.15 to 0.49) |
|  | 6 | 136 | 77117 | 0.35 (0.23 to 0.52) |
|  | 9 | 181 | 73775 | 0.40 (0.29 to 0.56) |
|  | 12 | 245 | 70127 | 0.42 (0.31 to 0.55) |
|  | 24 | 422 | 55434 | 0.61 (0.50 to 0.75) |
|  | 48 | 539 | 27023 | 0.66 (0.55 to 0.79) |
| Hospital admission for | |  |  |  |
| All causes | 3 | 6150 | 80959 | 0.70 (0.66 to 0.73) |
|  | 6 | 10365 | 78654 | 0.70 (0.67 to 0.73) |
|  | 9 | 13796 | 76199 | 0.71 (0.68 to 0.74) |
|  | 12 | 16315 | 73379 | 0.76 (0.73 to 0.79) |
|  | 24 | 22287 | 61413 | 0.86 (0.83 to 0.89) |
|  | 48 | 19543 | 34141 | 0.94 (0.89 to 0.98) |
| Cardiovascular disease | 3 | 1336 | 80421 | 0.68 (0.61 to 0.76) |
|  | 6 | 2277 | 77498 | 0.71 (0.65 to 0.77) |
|  | 9 | 2973 | 74393 | 0.74 (0.69 to 0.80) |
|  | 12 | 3628 | 70983 | 0.77 (0.72 to 0.83) |
|  | 24 | 5337 | 57038 | 0.89 (0.84 to 0.94) |
|  | 48 | 5388 | 29058 | 0.98 (0.92 to 1.04) |
| Respiratory disease | 3 | 1437 | 80375 | 0.64 (0.57 to 0.71) |
|  | 6 | 2305 | 77417 | 0.69 (0.63 to 0.75) |
|  | 9 | 3014 | 74280 | 0.74 (0.68 to 0.79) |
|  | 12 | 3583 | 70804 | 0.76 (0.71 to 0.81) |
|  | 24 | 5080 | 56716 | 0.86 (0.81 to 0.91) |
|  | 48 | 4975 | 28636 | 0.95 (0.89 to 1.02) |
| Primary care diagnosis of: | |  |  |  |
| Myocardial infarction | 3 | 70 | 78786 | 0.40 (0.24 to 0.68) |
|  | 6 | 110 | 75660 | 0.67 (0.45 to 0.98) |
|  | 9 | 146 | 72335 | 0.70 (0.50 to 0.97) |
|  | 12 | 177 | 68706 | 0.81 (0.60 to 1.09) |
|  | 24 | 280 | 54118 | 0.91 (0.72 to 1.16) |
|  | 48 | 317 | 26134 | 1.03 (0.82 to 1.30) |
| Chronic obstructive | 3 | 674 | 75336 | 0.67 (0.57 to 0.79) |
| pulmonary disease | 6 | 868 | 72390 | 0.71 (0.62 to 0.82) |
|  | 9 | 1070 | 69269 | 0.77 (0.68 to 0.88) |
|  | 12 | 1237 | 65816 | 0.80 (0.71 to 0.90) |
|  | 24 | 1682 | 51966 | 0.88 (0.80 to 0.97) |
|  | 48 | 1652 | 25266 | 0.98 (0.89 to 1.09) |

## Supplementary Table S5: Adjusted Relative Outcome Frequency Among Patients Treated With Varenicline or Nicotine Replacement Therapy Using Propensity score Methods. Follow-up at 3, 6, 9, 12, 24, 48 Months. Non-imputed data.

|  | Follow-up | Number of | Percentage difference |
| --- | --- | --- | --- |
|  | length | patients | (95% confidence intervals) |
| Outcome |  | N | Fully adjusted |
| Number of GP visits | 3 | 80,185 | 11.10 (6.63 to 15.76) |
|  | 6 | 77,005 | 2.86 (-0.74 to 6.59) |
|  | 9 | 73,617 | -0.18 (-3.35 to 3.08) |
|  | 12 | 69,909 | -1.37 (-4.29 to 1.63) |
|  | 24 | 55,032 | -1.98 (-4.53 to 0.63) |
|  | 48 | 26,462 | -1.15 (-4.04 to 1.82) |
| Number of | 3 | 81,840 | -7.20 (-8.23 to -6.17) |
| hospitalizations | 6 | 80,493 | -11.05 (-12.36 to -9.73) |
|  | 9 | 78,935 | -13.52 (-14.99 to -12.03) |
|  | 12 | 76,990 | -12.89 (-14.49 to -11.26) |
|  | 24 | 67,202 | -9.97 (-11.97 to -7.93) |
|  | 48 | 39,405 | -5.30 (-8.21 to -2.29) |
| Number of | 3 | 80,421 | -1.83 (-2.36 to -1.30) |
| hospitalizations | 6 | 77,498 | -2.88 (-3.59 to -2.17) |
| for respiratory disease | 9 | 74,393 | -3.32 (-4.15 to -2.49) |
|  | 12 | 70,983 | -3.62 (-4.57 to -2.66) |
|  | 24 | 57,038 | -2.93 (-4.38 to -1.46) |
|  | 48 | 29,058 | -1.11 (-3.98 to 1.84) |
| Number of | 3 | 80,375 | -2.27 (-2.82 to -1.71) |
| hospitalizations | 6 | 77,417 | -3.13 (-3.84 to -2.43) |
| for cardiovascular | 9 | 74,280 | -3.40 (-4.22 to -2.56) |
| disease | 12 | 70,804 | -3.85 (-4.79 to -2.90) |
|  | 24 | 56,716 | -3.57 (-4.94 to -2.18) |
|  | 48 | 28,636 | -2.36 (-5.10 to 0.47) |

## Supplementary Table S6: Means and bias of baseline covariates before and after propensity score matching.

|  |  | Mean | |  | | % reduction | |
| --- | --- | --- | --- | --- | --- | --- | --- |
|  |  | Varenicline | NRT | %bias | bias | |  |
| History | Unmatched | 3298 | 3143 | 7.9 |  | |  |
|  | Matched | 3298 | 3303 | -0.2 | 97 | |  |
| Male | Unmatched | 0.50 | 0.46 | 7.8 |  | |  |
|  | Matched | 0.50 | 0.50 | 1.1 | 86.3 | |  |
| Age (years) | Unmatched | 44.4 | 46.7 | -15.6 |  | |  |
|  | Matched | 44.4 | 44.5 | -0.4 | 97.3 | |  |
| BMI (kg/m2) | Unmatched | 26.5 | 26.4 | 0.9 |  | |  |
|  | Matched | 26.5 | 26.5 | 0.2 | 80.9 | |  |
| Missing BMI | Unmatched | 0.14 | 0.13 | 2.2 |  | |  |
| Prescription year: | Matched | 0.14 | 0.14 | -0.1 | 96.1 | |  |
| 2007 | Unmatched | 0.11 | 0.23 | -34.4 |  | |  |
|  | Matched | 0.11 | 0.11 | -1.8 | 94.7 | |  |
| 2008 | Unmatched | 0.14 | 0.16 | -4 |  | |  |
|  | Matched | 0.14 | 0.14 | 1.4 | 65.2 | |  |
| 2009 | Unmatched | 0.16 | 0.15 | 2.6 |  | |  |
|  | Matched | 0.16 | 0.16 | 0.7 | 70.7 | |  |
| 2010 | Unmatched | 0.18 | 0.13 | 14.2 |  | |  |
|  | Matched | 0.18 | 0.19 | -1.2 | 91.3 | |  |
| 2011 | Unmatched | 0.16 | 0.11 | 15.9 |  | |  |
|  | Matched | 0.16 | 0.17 | -0.6 | 96.3 | |  |
| 2012 | Unmatched | 0.13 | 0.08 | 14.6 |  | |  |
|  | Matched | 0.13 | 0.12 | 0.7 | 95.4 | |  |
| 2013 | Unmatched | 0.09 | 0.06 | 14.3 |  | |  |
|  | Matched | 0.09 | 0.09 | 1.3 | 90.7 | |  |
| 2014 | Unmatched | 0.02 | 0.01 | 5.3 |  | |  |
|  | Matched | 0.02 | 0.02 | 0 | 99.6 | |  |
| Misuses alcohol | Unmatched | 0.05 | 0.07 | -9.6 |  | |  |
|  | Matched | 0.05 | 0.05 | 0.3 | 96.5 | |  |
| Misuses drugs | Unmatched | 0.02 | 0.03 | -7.5 |  | |  |
|  | Matched | 0.02 | 0.02 | 0 | 99.4 | |  |
| Index of Multiple Deprivation | Unmatched | 11.2 | 11.7 | -9.2 |  | |  |
|  | Matched | 11.2 | 11.3 | -1.8 | 80.3 | |  |
| Missing IMD | Unmatched | 0.001 | 0.001 | -0.1 |  | |  |
| Prescribed: | Matched | 0.001 | 0.001 | 0.2 | -60.6 | |  |
| Hypnotics | Unmatched | 0.17 | 0.20 | -8.9 |  | |  |
|  | Matched | 0.17 | 0.17 | -0.4 | 95.4 | |  |
| Antipsychotics | Unmatched | 0.14 | 0.20 | -14.9 |  | |  |
|  | Matched | 0.14 | 0.14 | -0.2 | 98.7 | |  |
| Antidepressants | Unmatched | 0.43 | 0.50 | -14.2 |  | |  |
|  | Matched | 0.43 | 0.44 | -1.6 | 88.5 | |  |
| Statins | Unmatched | 0.15 | 0.19 | -12.1 |  | |  |
|  | Matched | 0.15 | 0.15 | -1.2 | 90.3 | |  |
| Antihypertensives | Unmatched | 0.17 | 0.21 | -10.3 |  | |  |
|  | Matched | 0.17 | 0.17 | -0.2 | 97.9 | |  |
| Diabetic medications | Unmatched | 0.06 | 0.09 | -10 |  | |  |
| Previously diagnosed with: | Matched | 0.06 | 0.07 | -0.7 | 92.5 | |  |
| Self-harm | Unmatched | 0.09 | 0.10 | -6 |  | |  |
|  | Matched | 0.09 | 0.09 | -0.7 | 88 | |  |
| Myocardial infarction | Unmatched | 0.02 | 0.03 | -9.1 |  | |  |
|  | Matched | 0.02 | 0.02 | -0.4 | 96 | |  |
| Chronic obstructive pulmonary | Unmatched | 0.06 | 0.08 | -7.9 |  | |  |
| disease | Matched | 0.06 | 0.06 | -0.6 | 92 | |  |
| Chronic disease (Charlson index) | Unmatched | 0.32 | 0.38 | -13.6 |  | |  |
|  | Matched | 0.32 | 0.32 | -0.6 | 95.5 | |  |
